# Why Members of the Dental Profession Can Not and Should Not Be Classed as Manufacturers

**Published:** 1921-02

**Authors:** 


### WHY MEMBERS OF THE DENTAL PROFESSION CAN NOT AND SHOULD NOT BE CLASSED AS MANUFACTURERS

Dr. R. G. McLaughlin, Contributing Editor of *Oral Health* and Professor of Ethics and Jurisprudence of The Royal College of Dental Surgeons, was invited to prepare a statement showing the essential difference between a business and a profession, and particularly regarding the right of local excise officers to ask menbers of the dental profession to pay a two per cent. tax upon prosthetic or other dental appliances made in their offices and essential to the service rendered their patients.

The statement prepared by Dr. McLaughlin is of such general interest to the profession that we publish the greater part of it in full:

''The accepted legal definition of a business man is one who is engaged in exchanging commodities for other commodities or for money. He is engaged in the work of buying and selling.

''A profession is a special calling which only those are allowed to follow who have conformed to a prescribed course of education and training, have passed successfully the necessary or prescribed examinations, are of good moral character, and have finally been licensed by special statute to practice in that profession.

''The law, as you see, draws a very clear line both legally and ethically between a business and a profession. The business man can not cross the line into the ranks of a profession, unless he first conforms to the legal requirements governing that profession; and the professional man can not commercialize the practice of his profession with impunity. He would be found guilty of unprofessional conduct, and liable to have his license suspended or revoked.

''The relation of a dental surgeon to his patient is not that of a seller and buyer, nor that of employer and employe; it is a trust of the utmost importance confided to the dentist by his patient, who entrusts his future comfort and health, and not infrequently life itself, to the professional knowledge, skill, good judgment and sincerity of the dentist. The patient does not come as a buyer, seeking to purchase something the dentist has to sell. * * * He is sick and suffering from the diseased condition in the oral cavity or that region, and he comes to the dentist for professional advice and services. He legally expects from the dentist, as a professional man, the necessary professional knowledge and skill to diagnose his trouble and prescribe the proper remedies, whether they be medicinal, surgical, mechanical, advisory, or all four combined.

''We must understand that dentistry and medicine are sister professions. We are engaged in the same great work of caring for the health and physical welfare of the public. As surgeons treating different parts of the same body, and restoring lost or damaged parts of the human anatomy—whether it be a lost eye, a lost tooth, a lost nose or a damaged arm—we are governed by the same ethical customs and legal rulings.

''If you class as a manufacturer the dental surgeon who replaces a lost or damaged tooth by an artificial one, then by the same reasoning the surgeon who replaces a lost eye by an artificial one, who makes a splint to bind up a broken bone, or who makes up a box of pills, must be classed as a manufacturer. In short, the dental surgeon and the general surgeon are ethically and legally on the same plane, and such a ruling as proposed applies to the one with the same force as it does to the other.

''In short, we must answer this question: Can a member of a profession which has been organized by special statute, and its membership rigidly restricted to those who have won their diplomas by a long, arduous course of study—can a legally qualified member of that profession—be he a dental surgeon or a general surgeon—while strictly following the practice of his profession, be legally called a manufacturer or a tradesman?

"The Supreme Court of Mississippi was asked to pass upon this point: the question before the court was whether a dentist's instruments were exempt from execution under a statute exempting the tools of a mechanic necessary for the carrying on of his trade. The Court said: 'We do not think that this provision can be extended to the description of instruments in question. A dentist can not be properly denominated a mechanic.'

"From the time of Federation, when the British North America act gave each province control of its own educational matters, every precaution has been taken in Ontario, by amendments and additions to the dental act, and by special by-laws, to prevent members of the profession from commercializing the practice of dentistry. Such restrictions and precautions were considered necessary in the public interest. It would be considered a misdemeanor today for any member of the dental profession in Ontario to so practice his profession or conduct his practice as a commercial concern. Some years ago, in the city of Toronto, an attempt was made to commercialize the profession by ordinary laymen establishing concerns known as dental parlors, etc., and hiring licensed dentists by the day or week to work for the patients. This matter was taken to the court, and the decision and opinion of Chief Justice Anglin is worthy of notice."—*Oral Health*, December, 1920.

